# Paracetamol, Ondansetron, Granisetron, Magnesium Sulfate and Lidocaine and Reduced Propofol Injection Pain

**DOI:** 10.5812/ircmj.16086

**Published:** 2014-03-05

**Authors:** Mohammad Alipour, Masoomeh Tabari, Masoomeh Alipour

**Affiliations:** 1 Department of Anesthesiology, Faculty of Medicine, Mashhad University of Medical Sciences, Mashhad, IR Iran

**Keywords:** Acetaminophen, Magnesium Sulfate, Ondansetron, Granisetron, Lidocaine

## Abstract

**Background:**

Propofol is a most widely used intravenous anesthetic drug. One of its most common complications is the pain upon injection; therefore, different methods, with various effects, have been proposed in order to alleviate the pain.

**Objectives:**

This study investigates the effects of paracetamol, ondansetron, granisetron, magnesium sulfate and lidocaine drugs on reducing the pain of propofol injection during anesthetic induction. Also, the hemodynamic changes will be analyzed.

**Patients and Methods:**

This is an interventional study containing 336 patients underwent elective orthopedic surgeries in Educational Hospitals of Mashhad University, using systematic sampling, the patients were divided into six groups. A 20-gauge needle was inserted into a venous vessel in the back of the hand and 100 cc of Ringer serum was injected into the vein, which was applied proximal to the injection site. Afterwards, paracetamol 2 mg/kg (group p), magnesium sulfate 2 mmol (group M), ondansetron 4 mg (group O), granisetron 2 mg (group G), lidocaine 40 mg (group L) and 5 cc saline (group S) were injected into the vessel, after 60 seconds, the tourniquet was opened. One quarter of the total dose of propofol (2.5 mg/kg) was injected with a flow rate of 4 mg/sec and then the injection pain was measured. Finally, the fentanyl (2 µg/kg), atracurium 0.5 mg/kg, and the remaining dose of propofol were injected and the vital signs were recorded before the administration of propofol and 1, 3, 5 and 10 minutes after the propofol injection.

**Results:**

The six groups did not significantly differ, regarding their gender, weight or age. Propofol injection pain was less in L and G groups, in comparison with the others (P ≤ 0.001). By analyzing the hemodynamic changes, it was observed that the least amount of change in mean arterial pressure was observed in the paracetamol group.

**Conclusions:**

The reduction of propofol injection pain was observed by using medications (in comparison with normal saline), but it was more significant in groups G and L. Moreover, Hypotension was higher in groups S and G and it was lessened in group P.

## 1. Background

Propofol, which is widely used due to its rapid onset and recovery, is the most common intravenous anesthetic dug used for the induction and maintenance of anesthesia. This medicine is classified as phenols, and is insoluble in water and prepared in oil emulsion. The common complications of propofol are the pain at the injection site and hypotension (1). Since propofol has high lipid solubility, it causes painful intravenous injections. In order to reduce the pain of this combination, which consists of long-chain triglyceride solution, a medium and long-chain fatty mixture is used. Propofol causes immediate pain because of the local vein stimulation, and also can lead to a delayed type of pain, after about 15 seconds, due to the activation of kallikrein and bradikinine. In some studies, the incidence of propofol injection pain has been estimated about 28 - 90% (2) and about 85% in another study (3). Pain severity in propofol injection, based on VAS pain measurement system (pain score ranged from zero to ten), has been reported 5.6 ± 3.2, which is indicating as severe pain (4).

Various techniques have been used in different studies in order to decrease the pain of propofol injection, including diluting propofol, cooling or heating, using larger vessels for injection or adding lidocaine. Also, The drugs which have been studied to decrease the pain included different doses of lidocaine (5-9), NSAIDs (5), metoclopramide (6), opiates such as morphine, meperidine, fentanyl (7) tramadol (2), thiopental sodium (4), nafamostatmesilate (10), ketamine (8, 11), ondansetron (12) granisetron (13, 14) magnesium sulfate (8, 15-18) and paracetamol (9, 19), have been associated with different results.

## 2. Objectives

We studied the effects of, paracetamol, lidocaine, magnesium sulfate, ondansetron and granisetron to prevent the propofol injection pain during anesthetic induction. The induced hemodynamic changes, is a common complication of the injection, which was also investigated, in order to get the best, cheapest, most available and the most suitable drug based on the patient’s condition.

## 3. Patients and Methods

This interventional prospective study was conducted as a clinical trial. The sampling method was based on either systematic or probability sampling and the patients were randomly classified into one of the six groups using block randomization, and randomized numerical table. Data was gathered using the field method, observation and the checklist. Using NCSS software package for sample size determination, PASS, and considering effect size of 0.2, power of 80 percent and type 1 error of 0.05, required sample size has been calculated 336 subjects. Inclusion criteria were patients registered in Mashhad hospitals, a popular city of Iran, who were candidates for elective orthopedic surgery with ASA class I and II, and also were 20 - 50 years old. On the other hand, exclusion criteria included allergy to propofol, thin veins in back of the hand, severe mental and neurological disorders, neuromuscular diseases, heart disease, convulsion, pregnancy and breast-feeding, and BMI above 30. The present study was approved by the Ethics Committee of Mashhad University of Medical Sciences, No. 89, 664 on September 07, 2011 and the informed consents were obtained from 336 selected patients, based on the sampling design by ASA I, II class. Patients were randomly divided into six groups of 56 members, using a random numerical table. Premedication was not prescribed. For all patients, a 20-gauge needle was inserted into a large venous vessel in the back of the hand, and they received 100 cc of Ringer serum. The pulseoxime try monitoring, blood pressure and heart rate of all patients were measured and tourniquet was applied proximal to the injection site in a way to only block the serum flow. The studied drug was prepared and encoded in 5 cc volume by the nurse who blinded to the study groups. Afterwards the studied drugs including paracetamol 2 mg/kg (group P), magnesium sulfate 2 mmol (group M), ondansetron 4 mg (group O), granisetron 2 mg (group G), lidocaine 40 mg (group L) and saline solution (group S) were injected with an equal volume of 5 cc, before propofol injection. After 60 seconds, the tourniquet was opened and one quarter of the total dose of propofol 2.5 mg/kg (Germany model, Hamburg, Fresenius Kabi) was injected with a flow rate of 4 mg/sec. Later, an anesthesiology resident who was unaware of the type of administered drug, asked the patient about the severity of the pain. The data were recorded based on a four-point scale (no = zero, low = 1, moderate = 2, severe = 3). Then, the induction of anesthesia was performed, using fentanyl 2 µg/kg, atracurium 0.5 mg/kg and the remaining dose of propofol. Vital signs before the injection of propofol and at minutes 1, 3, 5 and 10 were recorded.

After determining the values of variables and the initial processing, the data were entered in SPSS software version 11.5. All the data were characterized using descriptive statistics including frequency tables, columnar graphs, frequency distributions and by calculating the statistical indices of central tendency and dispersion. Considering the normal distribution of data and the statistical analysis, the chi-square for categorical data and one-way ANOVA test for continuous data were used to evaluate the objectives and hypotheses of this research. P value < 0.05 was considered as significant.

## 4. Results

**Figure 1.**
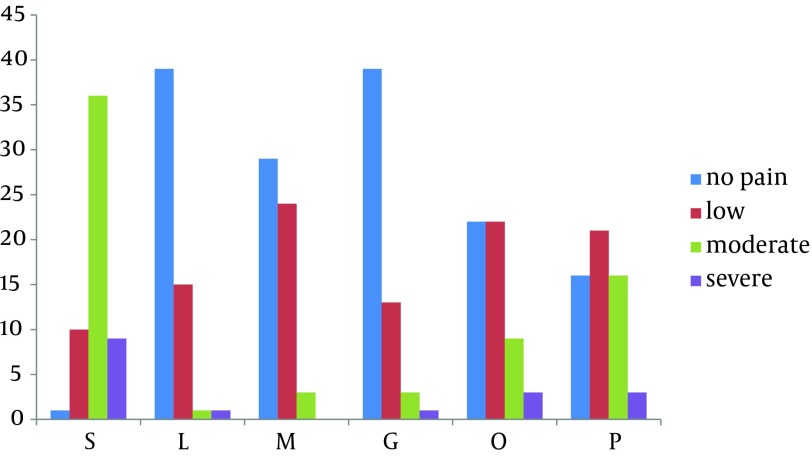
Comparison of the Incidence of Pain in the Studied Groups

According to the statistical results, the minimum age of patients was 20 years old and the maximum age was 50. The mean age was 40.13 years with a standard deviation of 10.18. Demographic characteristics of the six groups of participants in terms of age, gender and weight did not differ (P value = 0.544, P value = 0.057 and P value = 0.402, respectively) (Table 1). The comparison of propofol injection pain, using the Chi square test between different groups indicates a significant difference. Thus, only one person (1.7%) in the saline group has no complaints of pain, while the number of patients without pain in lidocaine group was 39 (69.64%), in magnesium sulfate was 29 (51.78%), in granisetron group was 39 (69.64%), in ondansetron group was 22 (39.23%) and in paracetamol group was 16 (28.57%), (P ≤ 0.001) (Table 2). Ascan be seen, the amount of pain reduction in two groups receiving lidocaine and granisetron was equal and higher than the other groups (Figure 1). 

**Table 1.** Distribution of Age and Gender in the Study Participants ^a^

	Group P	Group O	Group G	Group M	Group L	Group S
**Age, y**	39.84 ± 10.34	39.12 ± 8.47	41.84 ± 10.37	41 ± 11.30	55.11 ± 48.40	71.8 ± 52.38
**Weight, kg**	70.82 ± 14.63	43.11 ± 20.69	06.14 ± 20.74	45.12 ± 25.70	90.13 ± 61.65	07.12 ± 61.70
**Male, No. (%)**	8.51 (29)	6.44 (25)	2.48 (27)	4.55 (31)	6.53 (30)	30.64 (36)
**Female, No. (%)**	2.48 (27)	4.55 (31)	8.51 (29)	6.44 (25)	6.46 (26)	7.35 (20)

^a^ All data are presented in Mean ± SD.

**Table 2.** Comparison of the Incidence of Pain in the Studied Groups

Severity of pain	Group P	Group O	Group G	Group M	Group L	Group S
**Without pain**	16	22	39	29	39	1
**Low**	21	22	13	24	15	10
**Moderate**	16	9	3	3	1	36
**Acute**	3	3	1	0	1	9

The hemodynamic status in studied groups demonstrated that in comparison with others, the paracetamol group had the minimal changes in mean arterial pressure, in minutes 1, 3 and 10 (minute 1, P ≤ 0.001, minute 3, P = 0.027 and minute 10, P = 0.011) (Table 3). On the other hand, the changes of the heart rate showed no significant difference among the groups, during the study (Table 4). 

**Table 3.** The Mean Arterial Pressure in Groups, in Different Times After Propofol Injection

Time	Group P	Group O	Group G	Group M	Group L	Group S
**Before induction**	79.101	23.100	79.109	90.109	95.103	73.100
**The first minute after the anesthetic induction**	12.90	83	7.95	48.94	20.87	36.82
**The third minute after the anesthetic induction**	59.90	86	61.93	61.88	20.86	96.84
**The fifth minute after the anesthetic induction**	66.91	3.95	09.96	45.96	46.91	64.91
**The tenth minute after the anesthetic induction**	32.97	2.102	39.101	36.100	00.96	91.103

**Table 4.** Heart Rate Changes in Each Group at Different Times After Propofol Injection

Time	Group P	Group O	Group G	Group M	Group L	Group S
**Before the anesthetic**	80.81	90.83	02.82	86.80	21.80	88.81
**One minute after the anesthetic induction**	59.74	46.73	50.74	82.74	20.75	79.72
**The third minute of the anesthetic induction**	46.72	32.71	70.72	45.70	59.71	89.69
**The fifth minute after the anesthetic induction**	82.72	30.73	80.72	07.69	84.70	62.72
**The tenth minute after the anesthetic induction**	87.75	73.74	77.73	82.70	50.72	25.75

## 5. Discussion

This study aimed to evaluate and compare the effects of five mentioned drugs on propofol injection pain during anesthetic inductions, as well as hemodynamic changes. The results of this study showed that pain caused by propofol had a significant difference between different groups. Thus, only one person in the group receiving saline solution (1.7%) had no complaint. In contrast, the number of people without pain was 39 in lidocaine patient group (69.64%), 29 in magnesium sulfate group (51.78%), 39 in granisetron group (69.64%), 22 in ondansetron group (39.28%) and 16 in paracetamol group (28.57%), (P ≤ 0.001). Jalota and colleagues, in a systematic and meta-analysis study examined 177 clinical trials involving 25, 260 adults. They reported that the propofol injection pain was 60 % (20). Picard et al. in a systematic study on 56 studies, including 6264 patients and 12 various drugs, showed that intravenous lidocaine (0.5 mg/kg), through the ligation of atourniquet on the forearm, 30 to 120 seconds before propofol injection, can reduce the pain up to 60% (15). In a study which was carried out in September 1999 by Ramer and colleagues, it was determined that the pain severity after propofol injection without anesthesia, was approximately 70%, which is not associated with the size of catheter (IV) or propofol infusion rate. In addition, the results of Radaideh’s study in 2007 on 200 patients, indicated that 4 cc of lidocaine 1% reduces the pain of propofol injection by 68 % (5). The results of these studies are consistent with our findings, indicating that lidocaine reduces the pain of propofol injection up to 69.64%. According to our results, 69.64% of patients in the granisetron group had no pain of propofol injection. In study of Ahsan Ahmad, granisetron along with vein ligation was effective in reducing the pain up to 85% (13). Prasad and Dubey’s study in 2003 was conducted on 150 patients, 5 cc of normal saline in group N/S, 40 mg of lidocaine in group L and 2 mg of granisetron in group G, were injected to a vein in the back of the hand. The tourniquet was opened after two minutes, and a quarter dose of propofol was injected. In this study, pain 82% reduced the lidocaine group and 78% in the granisetron group (14), which is similar to our findings.

The results of Zahedi et al. showed that ondansetron reduced the aforementioned pain in 57.8% of the patients (12), which is approximately similar to our results (39.28%). According to our results, Paracetamol reduces propofol injection pain up to 28.57%, which is slightly different from the results of other studies. In Radaideh’s study, 54% of the paracetamol group had no pain (5). Borazan et al. investigated the effects of lidocaine with different doses of paracetamol on the reduction of propofol injection pain. P 0.5, P1 and P2 groups were administered paracetamol 0.5 mg, 1 mg and 2 mg/kg of body weight, respectively. The L 0.5 group was administered lidocaine 0.5 mg per kg of body weight. In group C (the control group) isotonic saline was injected into the veins, and propofol injection pain was recorded after one minute. The pain was 76% in the control group, 26% in the lidocaine group, 40% in P 0.5 group (0.5 mg/kg paracetamol), 24% in P1 group (1 mg/kg paracetamol) and 8% in P2 group (2 mg/kg paracetamol). Finally, Borazan concluded that 2 mg/kg of paracetamol was more effective than lidocaine and the lower doses of paracetamol (19). Canbay O et al. in 2008 conducted a similar research to reduce the propofol injection pain by using 40 mg of lidocaine and 50 mg of intravenous acetaminophen. They recorded the pain up to 64% in the control group, 8% in lidocaine group and 22% in the paracetamol group. They concluded that acetaminophen is effective in reducing propofol injection pain but not as much as lidocaine, which is similar to our results (9). Our results indicated that by using magnesium sulfate therapy, 51.78% of patients had no pain complaints. In a study by Turanand Memis, in 2002 on 100 patients, the effect of magnesium sulfate was compared with normal saline. The results proved that in magnesium sulfate group, propofol injection pain was non-existent in 64% of cases (16). In another study by Honarmandand Safavi on 200 patients with ASA class I, II, III, the effects of magnesium sulfate, ketamine, lidocaine and saline were compared. The pain was reported 88% in the control group, 34% in magnesium sulfate group, 28% in the ketamine and 18% in the lidocaine group. This study shows that in magnesium sulfate group, 66% of patients had no pain (8). The effect of magnesium sulfate in these two studies was slightly higher than our study, which could be due to the higher dose of magnesium sulfate used, compared with our study. In the study of Shoaybi and colleagues (2008) on 100 ASA I, II patients, the first group received magnesium sulfate 10% in one hand and the same volume of normal saline in the other hand. In group 2, magnesium sulfate 10% was injected in one hand and lidocaine 1% into the other one, and after 30 seconds, 2 mL of propofol 1% was injected simultaneously in the back of both hands. They concluded that pretreatment with magnesium sulfate have not resulted to any significant change of propofol injection pain in comparison with lidocaine. Although, both of them are more effective than normal saline (17). Agarwal et al. studied the effects of magnesium sulfate with lidocaine. They concluded that 40 mg lidocaine and 1 mg magnesium sulfate were equally effective in lessening the injection pain of propofol (42% and 30%, respectively) but 31% of patients in magnesium sulfate group had pain during the injection of magnesium sulfate (before the injection of propofol). As a result, magnesium sulfate was not recommended (18). Our results also showed that even though there is no significant difference between lidocaine and magnesium sulfate, lidocaine is more effective. In Agarwal’s study, the pain of magnesium injection is probably due to the low volume of drug (2 mL). The results of this study indicated that all of five drugs had almost similar effects in reducing the propofol injection pain compared with the control group, therefore, these results were in accordance with the results of other investigations. The effects of lidocaine and granisetron are similar (69.64%) and higher than the other groups. The effects of magnesium sulfate (51.78%), ondansetron (39.28%) and paracetamol (28.57%) are less than the lidocaine and granisetron, respectively. According to the results, we conclude that the obvious effects of granisetron and lidocaine in reducing the pain of propofol injection are similar and can appropriately prevent other related side effects. In the present study, the reduction of mean arterial pressure was less in the paracetamol group, compared with the other ones. Therefore, by considering the benefits and disadvantages of other medicines, it is suggested to use paracetamol as a premedication to reduce the propofol injection pain, for patients predisposed to hypotension. A detailed investigation of the effects of other drugs, such as opioids and corticosteroids to reduce the propofol injection pain, is recommended. Also, different doses of opioids along with hemodynamic and their side effects should be evaluated, in order to find the proper premedication to reduce the side effects caused by propofol injection, in various conditions.

## References

[1] Gupta S, Ravalia A, Jonnada HR (2001). Pain on injection with propofol.. Anaesthesia..

[2] Wong WH, Cheong KF (2001). Role of tramadol in reducing pain on propofol injection.. Singapore Med J..

[3] Morton NS, Johnston G, White M, Marsh BJ (1992). Propofol in paediatric anaesthesia.. Pediatr Anesth..

[4] Haugen RD, Vaghadia H, Waters T, Merrick PM (1995). Thiopentone pretreatment for propofol injection pain in ambulatory patients.. Can J Anaesth..

[5] El-Radaideh KM (2007). [Effect of pretreatment with lidocaine, intravenous paracetamol and lidocaine-fentanyl on propofol injection pain. Comparative study.. Rev Bras Anestesiol..

[6] Movafegh A (2003). A Comparison Of Metoclopramide And Lidocaine For Preventing Pain On Injection Of Propofol.. Tehran Univ Med J..

[7] Pang WW, Mok MS, Huang S, Hwang MH (1998). The analgesic effect of fentanyl, morphine, meperidine, and lidocaine in the peripheral veins: a comparative study.. Anesth Analg..

[8] Honarmand A, Safavi M (2008). Magnesium sulphate pretreatment to alleviate pain on propofol injection: A comparison with ketamine or lidocaine.. Acute Pain..

[9] Canbay O, Celebi N, Arun O, Karagoz AH, Saricaoglu F, Ozgen S (2008). Efficacy of intravenous acetaminophen and lidocaine on propofol injection pain.. Br J Anaesth..

[10] Iwama H, Nakane M, Ohmori S, Kaneko T, Kato M, Watanabe K (1998). Nafamostat mesilate, a kallikrein inhibitor, prevents pain on injection with propofol.. Br J Anaesth..

[11] Zhao GY, Guo Y, Bao SM, Meng LX, Zhang LH (2012). Prevention of propofol-induced pain in children: pretreatment with small doses of ketamine.. J Clin Anesth..

[12] Zahedi H, Maleki A, Rostami G (2012). Ondansetron pretreatment reduces pain on injection of propofol.. Acta Med Iran..

[13] Ahmed A, Sengupta S, Das T, Rudra A, Iqbal A (2012). Pre-treatment with intravenous granisetron to alleviate pain on propofol injection: A double-blind, randomized, controlled trial.. Indian J Anesth..

[14] Dubey PK, Prasad SS (2003). Pain on injection of propofol: the effect of granisetron pretreatment.. Clin J Pain..

[15] Picard P, Tramer MR (2000). Prevention of pain on injection with propofol: a quantitative systematic review.. Anesth Analg..

[16] Memis D, Turan A, Karamanlioglu B, Sut N, Pamukcu Z (2002). The use of magnesium sulfate to prevent pain on injection of propofol.. Anesth Analg..

[17] Shoaybi G, Soltanimohammadi S, Rajabi M (2008). The effect of Magnesium sulfate on reducing Propofol injection pain in elective surgeries.. Tehran Univ Med J..

[18] Agarwal A, Dhiraj S, Raza M, Pandey R, Pandey CK, Singh PK (2004). Vein pretreatment with magnesium sulfate to prevent pain on injection of propofol is not justified.. Can J Anaesth..

[19] Borazan H, Erdem TB, Kececioglu M, Otelcioglu S (2010). Prevention of pain on injection of propofol: a comparison of lidocaine with different doses of paracetamol.. Eur J Anaesthesiol..

[20] Jalota L, Kalira V, George E, Shi YY, Hornuss C, Radke O (2011). Prevention of pain on injection of propofol: systematic review and meta-analysis.. BMJ..

